# Modulating mitochondrial dynamics ameliorates left ventricular dysfunction by suppressing diverse cell death pathways after diabetic cardiomyopathy

**DOI:** 10.7150/ijms.98065

**Published:** 2024-09-03

**Authors:** Fumin Zhi, Xiangyi Pu, Wei Wei, Li Liu, Chunyan Liu, Ye Chen, Xing Chang, Hongtao Xu

**Affiliations:** 1 First Affiliated Hospital, Heilongjiang University of Chinese Medicine, Harbin 150040, China.; 2 Guang'anmen Hospital, China Academy of Chinese Medical Sciences, Beijing, 100053, China.; 3 Heilongjiang Forest Industry General Hospital, Beijing, 100053, Harbin 150000, China.

**Keywords:** mitochondrial fission, mitochondrial fusion, cardiomyocyte apoptosis, diabetic cardiomyopathy

## Abstract

Diabetic cardiomyopathy (DCM) triggers a detrimental shift in mitochondrial dynamics, characterized by increased fission and decreased fusion, contributing to cardiomyocyte apoptosis and cardiac dysfunction. This study investigated the impact of modulating mitochondrial dynamics on DCM outcomes and underlying mechanisms in a mouse model. DCM induction led to upregulation of fission genes (Drp1, Mff, Fis1) and downregulation of fusion genes (Mfn1, Mfn2, Opa1). Inhibiting fission with Mdivi-1 or promoting fusion with Ginsenoside Rg1 preserved cardiac function, as evidenced by improved left ventricular ejection fraction (LVEF), fractional shortening (FS), and E/A ratio. Both treatments also reduced infarct size and attenuated cardiomyocyte apoptosis, indicated by decreased caspase-3 activity. Mechanistically, Mdivi-1 enhanced mitochondrial function by improving mitochondrial membrane potential, reducing reactive oxygen species (ROS) production, and increasing ATP generation. Ginsenoside Rg1 also preserved mitochondrial integrity and function under hypoxic conditions in HL-1 cardiomyocytes. These findings suggest that restoring the balance of mitochondrial dynamics through pharmacological interventions targeting either fission or fusion may offer a promising therapeutic strategy for mitigating MI-induced cardiac injury and improving patient outcomes.

## Introduction

Diabetic cardiomyopathy (DCM) initiates a series of events leading to the loss of cardiomyocytes, the heart's contractile cells. Traditionally, necrosis, characterized by uncontrolled cell rupture, was considered the primary mode of cell death in DCM 1-3. However, recent studies have revealed a more complex scenario. Emerging evidence indicates that regulated cell death (RCD) pathways significantly contribute to DCM-induced cardiomyocyte loss 4, 5. These pathways, which include apoptosis, necroptosis, and ferroptosis, exhibit unique molecular signatures and morphological characteristics 6, 7. Apoptosis is a tightly regulated process involving the activation of caspases, proteases that dismantle cellular components. Apoptotic cardiomyocytes have been detected in the infarct border zone, suggesting a role in limiting infarct expansion 8, 9. Necroptosis, an inflammatory form of cell death, is triggered by receptor-interacting protein kinases (RIPKs). It has been implicated in both ischemic and reperfusion injuries, highlighting its potential as a therapeutic target 10, 11. Ferroptosis, an iron-dependent form of cell death characterized by lipid peroxidation, has been associated with oxidative stress in DCM. Targeting ferroptosis may offer a novel cardioprotective strategy^12^, ^13^.

These cell death pathways do not function in isolation. Interactions between these pathways and other cellular processes, such as autophagy and mitochondrial dysfunction, have been observed 14, 15. For example, inhibiting apoptosis can lead to increased necroptosis, suggesting a compensatory mechanism 13, 16. Understanding these complex interactions is crucial for developing effective therapies. The identification of RCD pathways in DCM has opened new therapeutic avenues 17. Targeting specific cell death modalities, either through pharmacological inhibition or genetic manipulation, may reduce infarct size and improve cardiac function 18. However, the optimal timing and combination of therapies remain to be determined.

Mitochondria, the cell's powerhouses, are crucial for cardiac function. Their dynamic nature, characterized by continuous fission (fragmentation) and fusion (elongation), is essential for maintaining cellular homeostasis 19. Recent research has revealed a critical link between mitochondrial dynamics and DCM, highlighting the potential for therapeutic interventions targeting these processes 20. During DCM, hyperglycemia insult triggers excessive mitochondrial fission, mediated by dynamin-related protein 1 (Drp1) activation 21. This fragmentation leads to the release of pro-apoptotic factors, impaired mitochondrial function, and ultimately, cardiomyocyte death 22. Studies have shown that inhibiting Drp1-mediated fission can attenuate DCM-induced cardiac injury, suggesting a promising therapeutic avenue. Conversely, promoting mitochondrial fusion through the upregulation of mitofusins (Mfn1 and Mfn2) has been shown to protect against DCM-induced damage 23. Fusion facilitates the exchange of mitochondrial contents, enabling the removal of damaged components and the restoration of mitochondrial function 24. However, the precise mechanisms underlying the protective effects of fusion remain to be fully elucidated. The intricate relationship between mitochondrial dynamics and DCM has spurred the development of novel therapeutic strategies. Modulating mitochondrial fission and fusion, as well as enhancing mitophagy, represent promising avenues for cardioprotection. However, further research is needed to optimize these interventions and translate them into clinical practice. The aim of our study is to investigate whether mitochondrial dynamics influence the progression of DCM by regulating cardiomyocyte apoptosis.

## Methods

### Ethical statement

Ethical approval for this study was granted by the Ethics Committee of Heilongjiang University of Chinese Medicine (2022-03-66). Experimental procedures were conducted in accordance with NIH guidelines. The study adhered to the Declaration of Helsinki and the institution's ethical guidelines.

### Animal Models

C57BL/6J wild-type mice (8 weeks old, male) received intraperitoneal injections of 50 mg/kg STZ for 5 consecutive days, following the AMDCC protocol. Diabetes was confirmed by fasting blood glucose levels (>16 mmol/L). All mice were euthanized at 32 weeks, and hearts and kidneys were collected for further analysis. To investigate the effects of mitochondrial fission and fusion, the mice were administered Mdivi-1 (1 mg/kg) or Ginsenoside Rg1 (10 mg/kg) prior to DCM induction.

### Histology

Heart tissues were fixed via immersion in a paraformaldehyde/glutaraldehyde solution. The fixed tissues were then processed for histology, involving paraffin embedding, sectioning at the Jefferson Anatomical Pathology Services Core Facility, and hematoxylin/eosin staining 25. High-quality digital images were captured using an EVOS digital microscope (M7000, Invitrogen). Myocardial structure was subsequently analyzed and quantified using FIJI (Image J) software.

### Echocardiography

Echocardiography on adult mice was conducted using a previously established method. For noninvasive M-mode echocardiography, mice were anesthetized with 2% isoflurane until they were unresponsive. The animals were then placed on the measurement platform, and the abdominal hair was gently removed using depilatory cream. A small amount of prewarmed ultrasound gel was evenly applied to the exposed abdomen, and the probe was carefully positioned in contact with the gel while gradually moving toward the skin to locate the beating heart. Videos were recorded once the heart was visualized on the screen. After completing the examination of all marked embryos, the mice were euthanized using CO_2_ followed by cervical dislocation 26. The abdominal skin and muscle layers were carefully incised, and each embryo was extracted from the uterine horn, labeled accordingly, and preserved for genotyping.

### Cell Culture and Reagents

To construct a high-glucose injury model in HL-1 cardiomyocytes, HL-1 cells are first cultured in Claycomb medium with supplements and maintained in a humidified incubator at 37°C with 5% CO_2_. High-glucose medium (25 mM D-glucose) and control normal-glucose medium (5.5 mM D-glucose) are prepared. HL-1 cells are seeded in culture dishes and allowed to adhere for 24 hours before being treated with either high-glucose or normal-glucose medium for 24 to 72 hours. To inhibit mitochondrial fission, cells were exposed to 5 nM Mdivi-1 for 6 hours. For the activation of mitochondrial fusion, cells were treated with 10 mM Ginsenoside Rg1 for 6 hours.

### ELISA

The wells of a microplate are coated with a capture antibody that specifically binds to the protein of interest. Non-specific binding sites within the wells are blocked to minimize false-positive signals. Samples or standards containing known quantities of the target protein are introduced into the wells, enabling the protein to be captured by the immobilized antibody. Unbound components are eliminated through washing procedures 27. A detection antibody, conjugated to an enzyme and specific to a distinct epitope of the target protein, is subsequently added. This antibody interacts with the captured protein, creating a sandwich complex. Surplus detection antibody is removed by additional washing steps. A substrate solution is then applied, which undergoes a reaction catalyzed by the enzyme linked to the detection antibody, resulting in a chromogenic change. The absorbance of the colored product is quantified using a microplate reader at a predetermined wavelength 28. The magnitude of the signal directly correlates with the quantity of target protein present in the sample. A standard curve is constructed using the absorbance values obtained from the standards with known target protein concentrations. The concentrations of the target protein in the experimental samples are subsequently interpolated from this standard curve based on their respective signal intensities.

### Reactive Oxygen Species (ROS) Measurement

Superoxide production was assessed using dihydroethidium (DHE) staining. Heart tissues were harvested, embedded in optimal cutting temperature (OCT) compound, and snap-frozen in liquid nitrogen. Sections of 5 μm thickness were prepared and incubated at 37°C in Krebs-Henseleit solution (KHS) containing 4 μM DHE for 30 minutes. Red fluorescence from DHE was visualized using a Leica confocal microscope (×40 objective). The integrated optical density (OD) of the stained heart tissues was quantified using ImageJ software, analyzing at least four sections per animal.

### Quantitative PCR (qPCR)

Total RNA was isolated from heart tissues or HL-1 cells using Trizol reagent (Invitrogen). For reverse transcription, 1 µg of RNA was used in a 20 µL reaction volume containing 4 µL iScript Reverse Transcription Supermix (Bio-Rad 1708841). The resulting cDNA was diluted threefold. PCR was conducted in duplicate for 40 cycles using 1 µL of cDNA in a 20 µL reaction volume, including 10 µL of PowerUP SYBR Green Master Mix (Thermo Fisher A25742). Real-time PCR was performed on a QuantStudio5 system (Thermo Fisher) with the following cycling conditions: 40 cycles of denaturation at 95°C for 15 seconds, followed by annealing and extension at 60°C for 60 seconds. Relative mRNA levels were quantified using the ΔCT method with Rpl7 as the reference gene.

### Western Blotting (WB)

Heart tissues were rinsed twice with cold PBS, and whole-cell lysates were prepared in RIPA buffer (50 mM Tris-HCl [pH 7.4], 150 mM NaCl, 1 mM EDTA, 1% Nonidet P40, 0.1% SDS, 1 mM dithiothreitol, 1:200 protease inhibitor cocktail [P8340, Sigma-Aldrich], and 1 mM PMSF). Western blot analysis was performed as previously described. Tubulin was used as a loading control and probed alongside the protein of interest 29. Tubulin immunoblots were often omitted for space considerations. Band intensities were quantified using ImageJ, with the mean intensity (integrated optical density) of control samples normalized to tubulin expression and set to 1. Fold changes were calculated relative to this baseline from at least five replicates 30.

### Statistical Analysis

Data are presented as the mean ± standard deviation (SD). Normality was assessed using the Shapiro-Wilk test and QQ plot inspection. Comparisons between two groups were made using a Student's t-test for normally distributed data with sample sizes greater than six, or a Mann-Whitney test for non-parametric data with sample sizes less than six. Comparisons among more than two groups were analyzed using one-way or two-way ANOVA with Sidak's post hoc test for multiple comparisons when significant interactions were observed. Mixed-effects analysis was employed to evaluate the effects of sex, diet, time, and genotype on perfusion recovery. A P-value of less than 0.05 was considered statistically significant. All analyses were performed using GraphPad Prism software (version 9.0) unless otherwise noted.

## Results

### Mitochondrial fission is activated whereas mitochondrial fusion is inhibited during DCM

**Figure 1:**
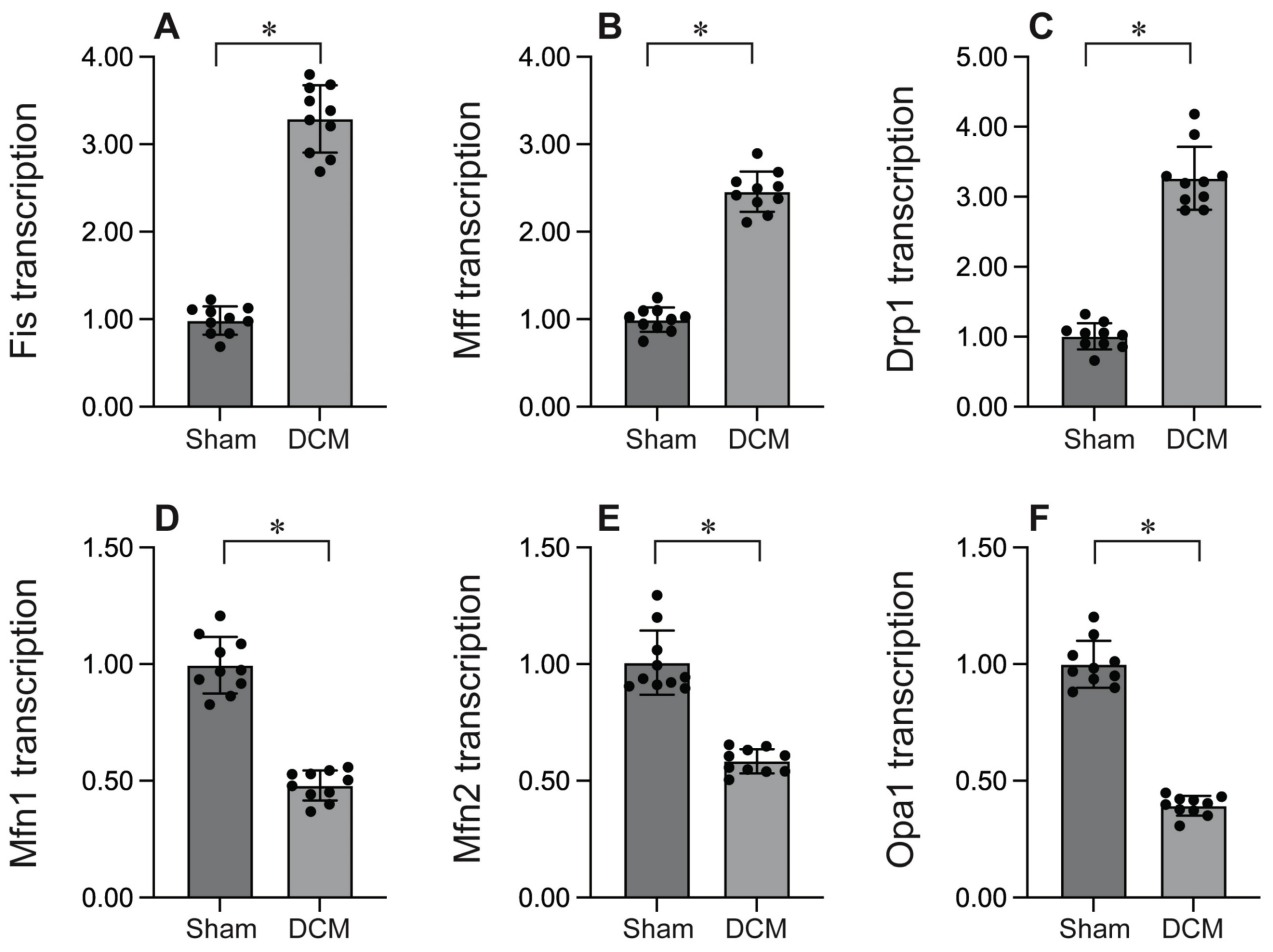
** Mitochondrial fission is activated whereas mitochondrial fusion is inhibited during DCM.** A-C. RNA was isolated from heart tissues after DCM. Then, the transcription of Fis1, Mff and Drp1 was determined by qPCR. D-F. RNA was isolated from heart tissues after DCM. Then, the transcription of Mfn1, Mfn2 and Opa1 was determined by qPCR. *p<0.05.

To investigate the impact of mitochondrial fission in the context of DCM, we employed qPCR to evaluate the expression of genes associated with mitochondrial fission. As depicted in Figure 1A-C, DCM enhanced the transcription of Drp1, Mff, and Fis1 compared to sham mice, indicating that mitochondrial fission is activated during DCM. Conversely, the expression of genes related to mitochondrial fusion, including Mfn1, Mfn2, and Opa1 (Figure 1D-F), was significantly suppressed following DCM, suggesting that mitochondrial fusion is inhibited by DCM. Collectively, our findings demonstrate that DCM activates mitochondrial fission while suppressing mitochondrial fusion.

### Inhibition of Mitochondrial Fission Attenuates Cardiac Injury and Cardiomyocyte Apoptosis

**Figure 2:**
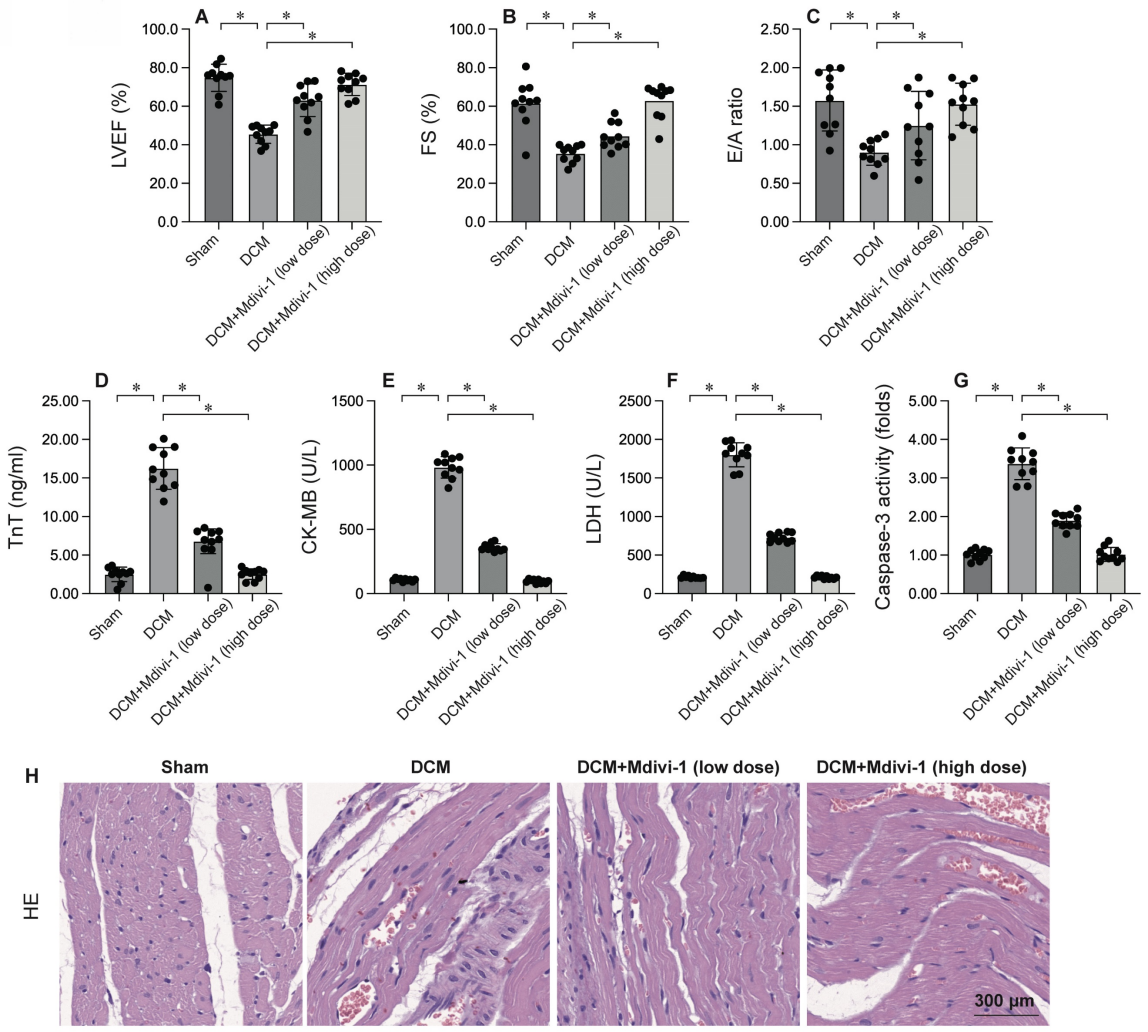
** Inhibition of Mitochondrial Fission Attenuates Cardiac Injury and Cardiomyocyte Apoptosis.** To investigate the effects of mitochondrial fission, the mice were administered low dose of Mdivi-1 (0.1 mg/kg) or high dose of Mdivi-1 (1 mg/kg) 12 hours prior to DCMinduction. A-C. Heart function was measured by echocardiography. D-F. ELISA kits were used to evaluate the levels of TnT, CK-MB and LDH in the serum of mice. G. The activity of caspase-3 was determined by an ELISA kit. H. HE staining of myocardium. *p<0.05.

To investigate the role of mitochondrial fission in DCM, Mdivi-1, a known inhibitor of mitochondrial fission, was administered prior to DCM induction. Cardiac function was subsequently assessed using echocardiography. As depicted in Figure 2A-C, compared to the sham group, DCM significantly compromised heart function, as evidenced by reductions in left ventricular ejection fraction (LVEF), fractional shortening (FS), and the E/A ratio. Remarkably, Mdivi-1 treatment preserved cardiac performance post-DCM (Figure 2A-C). Additionally, serum samples were collected from mice to measure levels of cardiac injury biomarkers, including troponin T (TnT), creatine kinase-MB (CK-MB), and lactate dehydrogenase (LDH). As shown in Figure 2D-F, these biomarkers were substantially elevated following DCM, but this increase was significantly attenuated by Mdivi-1. Collectively, these findings confirm that inhibiting mitochondrial fission protects the heart from DCM-induced damage (Figure 2D-F). To further explore the role of mitochondrial fission in cardiomyocyte apoptosis, caspase-3 activity was assessed using ELISA. Results demonstrated a significant increase in caspase-3 activity in heart tissues post-DCM (Figure 2G), which was notably reversed by Mdivi-1. HE staining showed that Mdivi-1 attenuates the hyperglycemia-mediated myocardial dysfunction (Figure 2H). In summary, inhibiting mitochondrial fission supports heart function and enhances cardiomyocyte survival.

### Activation of Mitochondrial Fusion is Linked to Cardiomyocyte Protection

**Figure 3:**
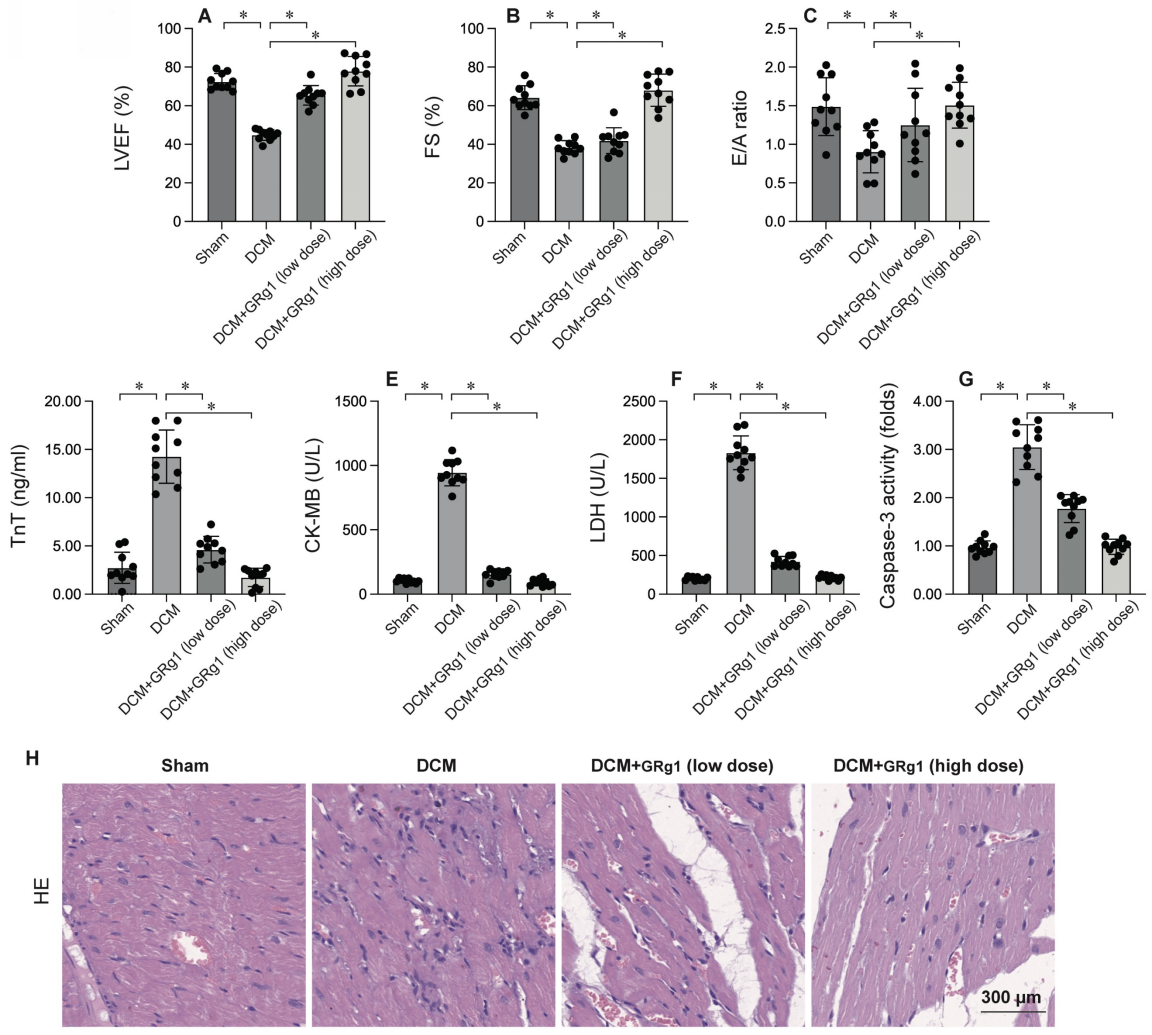
** Activation of Mitochondrial Fusion is Linked to Cardiomyocyte Protection.** To investigate the effects of mitochondrial fusion, the mice were administered low dose of Ginsenoside Rg1 (Grg1, 1mg/Kg) or high dose of Ginsenoside Rg1 (Grg1, 10 mg/kg) 12 hours prior to DCM induction. A-C. Heart function was measured by echocardiography. D-F. ELISA kits were used to evaluate the levels of TnT, CK-MB and LDH in the serum of mice. G. The activity of caspase-3 was determined by an ELISA kit. H. HE staining of myocardium. *p<0.05.

To elucidate the role of mitochondrial fusion in DCM, Ginsenoside Rg1, an agonist of mitochondrial fusion, was administered prior to DCM induction. Cardiac function was evaluated via echocardiography. As illustrated in Figure 3A-C, DCM significantly impaired heart function compared to the sham group, as indicated by decreased LVEF, FS, and E/A ratios. Interestingly, Ginsenoside Rg1 treatment preserved cardiac performance post-DCM. Moreover, serum samples were analyzed for cardiac injury biomarkers, such as TnT, CK-MB, and LDH. As shown in Figure 3D-F, these biomarkers were markedly elevated after DCM, but this elevation was significantly inhibited by Ginsenoside Rg1. These results collectively suggest that activating mitochondrial fusion protects the heart against DCM-induced injury. To further investigate the role of mitochondrial fusion in cardiomyocyte apoptosis, caspase-3 activity was measured using ELISA (Figure 3G). The results indicated a significant increase in caspase-3 activity in heart tissues post- DCM, which was effectively reversed by Ginsenoside Rg1. HE staining showed that activation of mitochondrial fusion attenuates the hyperglycemia-mediated myocardial dysfunction (Figure 3H). Overall, our findings demonstrate that activating mitochondrial fusion maintains myocardial function and cardiomyocyte viability.

### Inhibition of Mitochondrial Fission Enhances Mitochondrial Function

**Figure 4:**
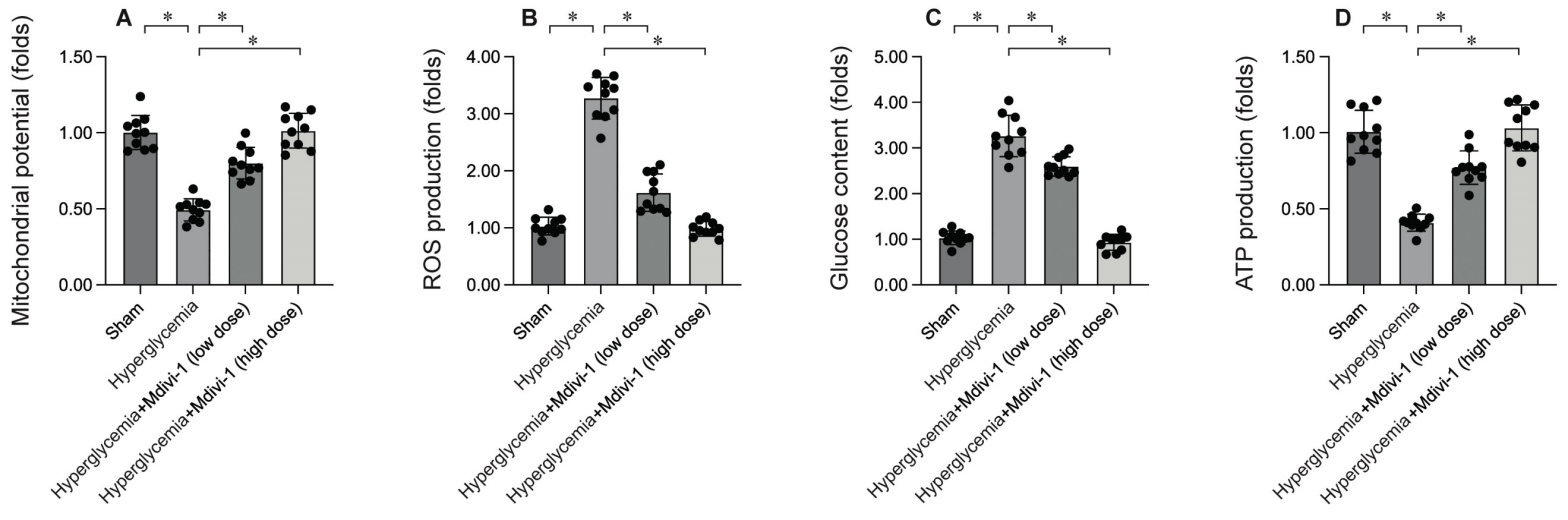
** Inhibition of Mitochondrial Fission Enhances Mitochondrial Function. HL-1 cells were treated with hypoxia for 6 hrs.** To inhibit the activity of mitochondrial fission, cells were treated with low dose of Mdivi-1 (0.5 nM) or high dose of Mdivi-1 (5 nM) 6 hours prior to high-glucose (25 mM D-glucose) treatment. A. Mitochondrial membrane potential was determined by immunofluorescence. B. ROS production was determined by DHE staining. C. The concentration of glucose in the medium was measured by ELISA. D. ATP production was determined by ELISA. *p<0.05.

To elucidate the molecular mechanisms by which inhibiting mitochondrial fission mitigates DCM-induced myocardial damage, mitochondrial function was assessed. HL-1 cells were subjected to 12 hours of hyperglycemia to simulate DCM in vitro. Immunofluorescence analysis revealed a significant reduction in mitochondrial membrane potential following hypoxia exposure (Figure 4A). Interestingly, treatment with Mdivi-1 restored mitochondrial membrane potential under hypoxic conditions. Additionally, hypoxia significantly increased reactive oxygen species (ROS) production in HL-1 cells (Figure 4B), an effect that was markedly inhibited by Mdivi-1. Mitochondria, the energy centers of cardiomyocytes, convert glucose into ATP. Under hypoxia, glucose concentration in the medium was significantly elevated (Figure 4C), accompanied by a decrease in ATP production (Figure 4D). Notably, Mdivi-1 treatment promoted glucose metabolism and enhanced ATP generation. These results collectively confirm that inhibiting mitochondrial fission improves mitochondrial function.

### Activation of Mitochondrial Fusion Maintains Mitochondrial Integrity

**Figure 5:**
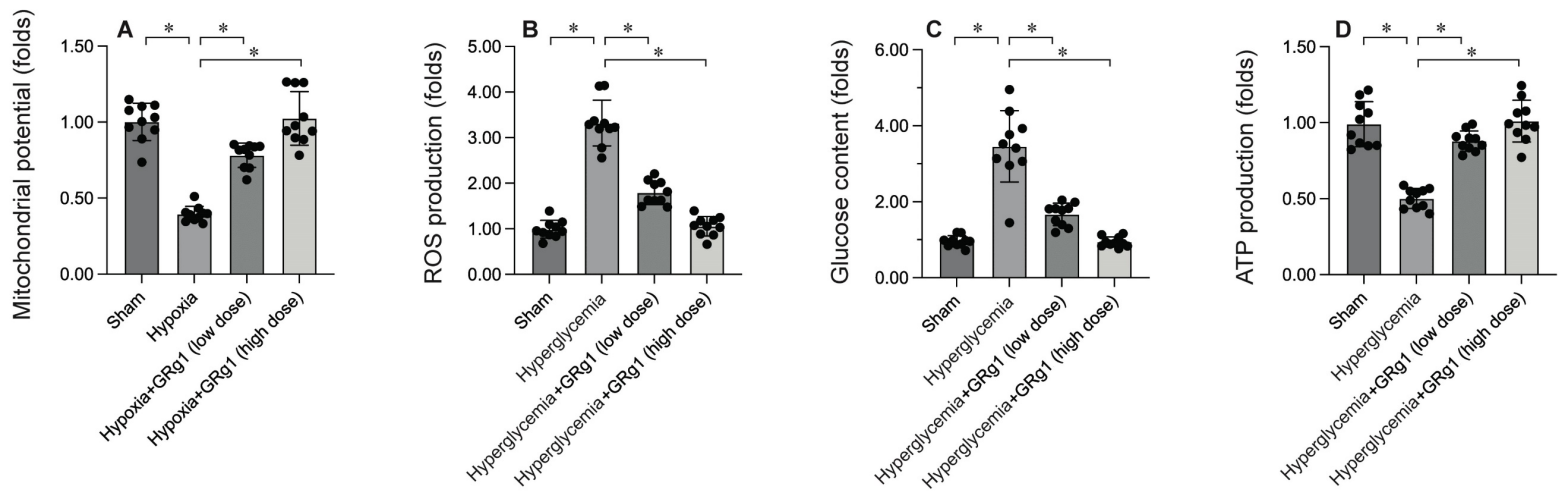
** Activation of Mitochondrial Fusion Maintains Mitochondrial Integrity.** To activate the activity of mitochondrial fusion, cells were treated with low dose of Ginsenoside Rg1 (GRg1, 1 mM) or high dose of Ginsenoside Rg1 (GRg, 10 mM) 6 hours prior to high-glucose (25 mM D-glucose) treatment. A. Mitochondrial membrane potential was determined by immunofluorescence. B. ROS production was determined by DHE staining. C. The concentration of glucose in the medium was measured by ELISA. D. ATP production was determined by ELISA. *p<0.05.

To uncover the molecular mechanisms by which activating mitochondrial fusion mitigates DCM-induced myocardial damage, mitochondrial function was evaluated. HL-1 cells were exposed to 12 hours of hypoxia to mimic DCM in vitro. Immunofluorescence analysis showed a significant reduction in mitochondrial membrane potential following hypoxia exposure (Figure 5A). Interestingly, Ginsenoside Rg1 treatment restored mitochondrial membrane potential under hyperglycemic conditions (Figure 5A). Additionally, hyperglycemia significantly increased ROS production in HL-1 cells (Figure 5B), an effect that was markedly inhibited by Ginsenoside Rg1. Mitochondria, the energy hubs of cardiomyocytes, convert glucose into ATP. Under hypoxia, glucose concentration in the medium was significantly elevated (Figure 5C), accompanied by a decrease in ATP production (Figure 5D). Notably, Ginsenoside Rg1 treatment promoted glucose metabolism and enhanced ATP generation. These findings collectively confirm that activating mitochondrial fusion improves mitochondrial function.

## Discussion

This study elucidates the pivotal role of mitochondrial dynamics in the pathogenesis of DCM. Our results reveal that DCM induces a profound shift in mitochondrial dynamics, characterized by upregulated expression of fission-related genes (Drp1, Mff, Fis1) and concomitant downregulation of fusion-related genes (Mfn1, Mfn2, Opa1). This dysregulation of mitochondrial dynamics contributes to cardiomyocyte apoptosis, as evidenced by elevated caspase-3 activity and impaired mitochondrial function.

Notably, our findings demonstrate that modulating mitochondrial dynamics can significantly impact the outcome of DCM. Pharmacological inhibition of mitochondrial fission with Mdivi-1 attenuated DCM-induced cardiac injury, preserved cardiac function, and reduced cardiomyocyte apoptosis. Conversely, promoting mitochondrial fusion with Ginsenoside Rg1 also conferred cardioprotection, improved cardiac function, and inhibited cardiomyocyte apoptosis. These results highlight the therapeutic potential of targeting mitochondrial dynamics in the treatment of DCM.

Mitochondrial fission, the process of mitochondrial fragmentation, has emerged as a pivotal factor in the pathogenesis of DCM. Although essential for maintaining mitochondrial quality control under physiological conditions, excessive or dysregulated fission can exacerbate cardiac injury during DCM 30-33. Investigations have consistently demonstrated increased mitochondrial fragmentation in ischemic cardiomyocytes, characterized by the accumulation of small, rounded mitochondria 31, 34. This fragmentation is mediated by the activation of dynamin-related protein 1 (Drp1), a GTPase that constricts and divides mitochondria 32, 35, 36. Fragmented mitochondria exhibit impaired respiratory capacity, reduced ATP production, and elevated reactive oxygen species (ROS) generation, contributing to cellular energy depletion and oxidative stress 37-39. Mitochondrial fission facilitates the release of pro-apoptotic factors, such as cytochrome c, from the intermembrane space, triggering programmed cell death 40, 41. Fragmented mitochondria can activate the NLRP3 inflammasome, leading to the release of pro-inflammatory cytokines and amplifying the inflammatory response 37, 42. The detrimental role of mitochondrial fission in DCM has spurred the development of therapeutic strategies aimed at inhibiting this process. Pharmacological inhibitors of Drp1, such as mdivi-1^31^, ^43^ and P110^44^-^46^, have shown promise in preclinical models of DCM, reducing infarct size and improving cardiac function.

Mitochondrial fusion, the process of mitochondrial elongation and networking, has emerged as a vital factor in the pathogenesis of DCM 47, 48. Although often overshadowed by the detrimental effects of excessive fission, recent research has revealed a protective role for mitochondrial fusion in mitigating ischemic injury 49. Despite the prevalence of mitochondrial fragmentation in DCM, studies have also observed compensatory upregulation of fusion proteins, such as mitofusins (Mfn1 and Mfn2) and optic atrophy 1 (Opa1) 12, 50, 51, suggesting an adaptive response aimed at preserving mitochondrial function and cellular viability. Fused mitochondria exhibit enhanced respiratory capacity, increased ATP production, and reduced ROS generation, mitigating cellular energy depletion and oxidative stress 51-53. Mitochondrial fusion inhibits the release of pro-apoptotic factors, such as cytochrome c, from the intermembrane space, thereby suppressing programmed cell death 54, 55. Investigations have demonstrated that enhancing mitochondrial fusion through genetic or pharmacological approaches can reduce infarct size, improve cardiac function, and promote cell survival in preclinical models of DCM 53, 56. The protective role of mitochondrial fusion in DCM has sparked interest in developing therapeutic strategies aimed at promoting this process. Small molecule agonists of Mfn1, such as S89, have shown promise in preclinical studies, demonstrating cardioprotective effects 57-59. Additionally, interventions targeting upstream regulators of mitochondrial dynamics, such as AMPK and sirtuins, may also offer therapeutic potential 35.

Mitochondria, the dynamic organelles responsible for cellular energy production, play a crucial role in determining cardiomyocyte viability during DCM. The intricate interplay between mitochondrial fragmentation and elongation governs cellular responses to ischemic injury, significantly influencing the extent of cardiomyocyte demise and subsequent cardiac dysfunction 60-62. In the ischemic environment of DCM, a surge in oxidative stress and calcium overload triggers excessive mitochondrial fragmentation, primarily mediated by the activation of dynamin-related protein 1 (Drp1) 63, 64. This disruption of mitochondrial integrity impairs ATP synthesis, exacerbates oxidative stress, and ultimately culminates in cardiomyocyte apoptosis and necrosis 38. Pharmacological inhibition of Drp1 has been shown to reduce infarct size and improve cardiac function, highlighting the detrimental consequences of unchecked mitochondrial fragmentation 65-67. In contrast, promoting mitochondrial elongation through the upregulation of mitofusins (Mfn1/2) and optic atrophy 1 (Opa1) has emerged as a promising cardioprotective strategy. Elongation enables the exchange of mitochondrial contents, facilitating the repair of damaged components and the restoration of bioenergetic function 52, 68, 69. This interconnected mitochondrial network acts as a buffer against ischemic stress, mitigating cardiomyocyte death and preserving cardiac function. The complex interplay between mitochondrial dynamics and cardiomyocyte fate in DCM has opened new avenues for therapeutic intervention 34, 70. Modulating mitochondrial fragmentation and elongation, as well as enhancing mitochondrial autophagy, represent promising strategies for cardioprotection. However, further research is necessary to optimize these interventions and translate them into clinical practice.

Our study provides mechanistic insights into the role of mitochondrial dynamics in cardiomyocyte survival. We found that inhibiting mitochondrial fission with Mdivi-1 enhanced mitochondrial function, as evidenced by improved mitochondrial membrane potential, reduced reactive oxygen species (ROS) production, and increased ATP generation. Conversely, promoting mitochondrial fusion with Ginsenoside Rg1 preserved mitochondrial integrity and function under hypoxic conditions. These results suggest that maintaining a delicate balance between mitochondrial fission and fusion is crucial for cardiomyocyte survival during DCM.

Our findings are consistent with previous studies demonstrating the detrimental effects of excessive mitochondrial fission in various cardiovascular diseases. However, our study goes further by showing that promoting mitochondrial fusion can also be a viable therapeutic strategy. This opens up new avenues for developing novel cardioprotective therapies targeting mitochondrial dynamics. Despite the promising results, our study has some limitations. First, we only investigated the effects of modulating mitochondrial dynamics in a mouse model of DCM. The translational potential of these findings to humans remains to be determined. Second, we only focused on the acute phase of DCM. The long-term effects of modulating mitochondrial dynamics on cardiac remodeling and function warrant further investigation.

In conclusion, our study provides compelling evidence that mitochondrial dynamics play a critical role in the pathophysiology of DCM. Modulating mitochondrial fission and fusion represents a promising therapeutic strategy for cardioprotection. Further research is needed to translate these findings into clinical practice and develop novel therapies for DCM.
